# Urinary equol levels are positively associated with urinary estradiol excretion in women

**DOI:** 10.1038/s41598-021-98872-2

**Published:** 2021-09-30

**Authors:** Tomoko Fujitani, Yukiko Fujii, Zhaoqing Lyu, Mariko Harada Sassa, Kouji H. Harada

**Affiliations:** 1 Department of Health and Environmental Sciences, Kyoto University Graduate School of Medicine, Kyoto, 606-8501 Japan; 2 Department of Pharmaceutical Sciences, Daiichi University of Pharmacy, Fukuoka, 815−8511 Japan

**Keywords:** Biomarkers, Medical research, Risk factors

## Abstract

Isoflavones found in soy products are a promising class of nutrients that may have a positive effect on human health. In particular, the phytoestrogen metabolite equol is associated with a reduced risk of developing female hormone-related diseases. However, the effect of equol on estrogen remains unclear. Equol can modify blood and urinary estradiol (E2) levels. The aim of this cross-sectional study was to examine the associations between urinary estrogen levels, equol levels, and equol production status in Japanese women. We analyzed urine samples from 520 women by gas chromatography-mass spectrometry. Urinary E2 and 4-hydroxylated E2 levels were higher in equol producers (EQP) than in non-EQPs (*P* < 0.0001 and *P*=0.00112, respectively). After adjusting for age and tobacco use by analysis of covariance, the association remained significant (β = 0.299, *P* < 0.0001). Analysis of covariance demonstrated that equol levels in urine were also positively associated with urinary E2 (β = 0.597, *P* < 0.0001). The log equol concentration showed a significant, but moderate, negative association with the serum E2 concentration (β = − 0.0225, *P* = 0.0462). Our findings suggest that equol may promote urinary E2 excretion and modify blood E2 levels in women.

## Introduction

The relationship between nutrients and cancer is an important public health issue. Estrogen can promote the development of several cancers, such as endometrial cancer and breast cancer; therefore, phytoestrogens are considered to be promising nutrients for cancer prevention. Indeed, the incidence of breast cancer in Asian countries is lower than that in Western countries^1^, which has led researchers to investigate lifestyles and dietary habits among Asian populations. The consumption of large amounts of soybeans or soybean products shows a significant association with a reduced risk of breast cancer^2^. Soybeans contain isoflavones, including daidzein and genistein^3^, which exhibit weak estrogenicity or anti-estrogenicity.

While daidzein is a major component of soy isoflavones, it can be metabolized to equol or *O*-desmethylangolensin (O-DMA) by human intestinal bacteria^4,5^. A previous study identified NADP(H)-dependent daidzein reductase from *Lactococcus*, converting daidzein to dihydrodaidzein (DHD)^6^. Moreover, DHD reductase, DHD racemase, tetrahydrodaidzein reductase were involved in equol production^7,8^. O-DMA is produced from DHD via C-ring cleavage by several bacteria^9^, but the enzyme remained unidentified. Individuals who can produce equol are known as equol producers (EQPs)^10^. Equol and daidzein are present at comparable levels in both the urine and blood of EQPs^11,12^ while in non-EQPs, the levels of equol are much lower than those of daidzein without association^13^. These features have prompted researchers to explore the prevalence of EQP status. The percentage of EQPs in Asian populations is 50–60%^14^, which was determined in sera by reversed-phase high-performance liquid chromatography-multiple reaction ion monitoring-mass spectrometry method with detection limit at 0.5 ng/mL. On the other hand, the percentage in the Western world is approximately 25–30%^15^, which was examined in urine by ultrahigh-pressure liquid chromatography with a detection limit at 2.93 nM. EQP status is related to age and dietary habit of taking isoflavone, but not sex^16^. Smoking is negatively associated with EQP status in women^17^.

Equol has the strongest estrogenic^18,19^ and antioxidative effects of all the isoflavones^10^. It has also been reported that equol was negatively associated with the risk of breast cancer in postmenopausal^4^ and premenopausal^20^ women, the incidence of hot flashes in perimenopausal and postmenopausal women^21^, and positively correlated with bone mineral density preservation^22^. These effects are likely mediated by estrogen receptors^23,24^. A study showed that blood estradiol (E2) levels are inversely correlated with urinary equol levels in postmenopausal women^25^, but it is still controversial^26,27^. In premenopausal women, isoflavone-rich soy products seemed inversely correlate with follicle-stimulating hormone (FSH) and luteinizing hormone (LH) and might mask the relationship between equol and E2^27,28^. Potential effects of equol on circulating hormones in reproductive women may influence their reproduction ability^29^. However, the mechanism of how equol reduces blood E2 levels remains unclear.

If urinary equol is negatively correlated to blood estradiol levels, there are several possibilities that equol reduces blood E2 levels. Equol may enhance metabolism of estrogens to hydroxylated estrogens, or equol may promote urinary excretion of estrogens. Therefore, we considered urinary and serum E2 as the main outcomes of such effects. In this observational study, we evaluated the associations between the concentrations of equol, estrogens, and their metabolites in the urine of postmenopausal and premenopausal Japanese women. In addition, the association between serum estradiol and urinary equol excretion was analyzed in select samples.

## Methods

### Study participants

In the original nationwide study, 13,910 participants aged 20–70 years were recruited through medical check-ups in 11 prefectures in Japan between 2000 and 2001^30^. The participants’ age, parity, smoking habits, present and past disease histories and menstrual status (regular cycles, irregular cycles, menopause, experienced gynecological surgery) were recorded using a self-report questionnaire. Body mass index (BMI) was obtained from participants in one study area, Kyoto (n = 109). A regular cycle was defined as menstrual cycles of 25–38 days. An irregular cycle was defined as menstrual cycles that were not regular (i.e., 25–38 days) in the previous 3 months. Menopause was defined as no menstruation for 12 months. Those who experienced gynecological surgery were categorized to eliminate the potential effects on estrogen levels. The questionnaire did not include anthropometry, dietary intakes such as soybean consumption, and length of menstrual cycle. Urine samples were collected at a time of no menstruation and were stored at − 30 °C for later analysis at the Kyoto University Human Specimen Bank^31,32^. The protocols for the specimen bank and for this study were in accordance with Ethical Guidelines for Medical and Health Research Involving Human Subjects of Japan and approved by the Ethics Committee of Kyoto University Graduate School of Medicine and Faculty of Medicine and Hospital (Latest approval number R1478-4 on May 19th, 2020, ‘Human exposure monitoring and risk assessment’). Written informed consent was obtained from all participants before sample collection.

For this study, 520 non-pregnant women from five prefectures recruited between November 2000 and December 2001 were randomly selected. A total of 100–110 samples per prefecture from the original participant group were analyzed for urinary isoflavone levels. No stratification and blocking for random selection were employed. All participants were of Asian ethnicity. Urinary isoflavones are biomarkers of blood isoflavone concentrations^17^. Urinary E2 levels were also analyzed to evaluate the association between equol and estrogen levels in the urine in a cross-sectional manner. The levels of estrogens other than E2 were measured in selected samples to evaluate the possible relationship between equol and estrogens. To confirm the relationship between equol and serum E2, an additional 100 paired urine and serum samples from the same original population were analyzed. Blood sampling was conducted by venipuncture in the morning under fasting conditions (more than 6 h) at the same institutions as urine samples. Serum was separated by centrifugation 30 min after sampling at room temperature and divided into polypropylene cryotubes. Serum samples were stored in –80 °C freezer.

### Analysis of isoflavones and estrogens

Target analytes in the urine were analyzed using a gas chromatography-mass spectrometer (model 6890GC/5973MSD; Agilent Technologies Japan Ltd., Tokyo, Japan) according to the extraction method described by Grace et al.^33^. Briefly, 0.5 mL of urine was mixed with 2,500 U of a glucuronidase/sulfatase solution and incubated for 12 h at 37 °C. The sample solution was then loaded onto a Sep-Pak Plus C18 solid-phase column (Waters, Milford, MA) and eluted with 3 mL of a 1:1 acetonitrile and ethyl acetate mixture. The eluates were dried under a nitrogen stream. Residues were derivatized to trimethylsilyl ethers in 100 μL of methyl *t*-butyl ether and 50 μL of *N,O*-bis(trimethylsilyl)trifluoroacetamide with 1% trimethylchlorosilane at 60 °C for 1 h. Derivatives were separated on a DB-5MS column (length 30 m, inner diameter 0.25 mm, film thickness 0.25 µm) (Supplementary Fig. 1), ionized by electron impact ionization, and quantified by single-ion monitoring using a mass spectrometer. We analyzed five estrogens (estrone (E1), estradiol (E2), estriol (E3), 4-hydroxy E1 (4-OHE1), and 4-hydroxy E2 (4-OHE2) and two isoflavones (daidzein and equol). 4-OHE1 is metabolized from E1, and 4-OHE2 is metabolized from E2. The detection limits for the analyses were 0.1 (4-OHE2) to 1 (E2) ng/mL for the estrogens, 20 ng/mL for equol, and 28 ng/mL for daidzein. Quantification was conducted with internal standard (d4-daidzein) and nine points calibration curves (0.1 ng/mL to 1000 ng/mL). Linearity of the calibration curves (*r*) ranged from 0.991 (E2) to 0.998 (daidzein). Samples that contained analytes over the range of calibration curve were analyzed again after dilution by distilled water. In every 10 samples, quality control sample was analyzed to confirmed validity of the analysis. Coefficient of variation in quality control samples were less than 9% for all analytes (N = 52). Serum E2 and progesterone were analyzed by ELISA in accordance with the manufacturer’s instructions (ADI-900-174 and ADI-900-011, Enzo Life Sciences, Inc., Farmingdale, NY).

To validate the stability of analytes in archived samples, urine samples obtained within one month (n = 30) were analyzed in the same method. We confirmed that distribution and trend of analytes were comparable between long-archived samples and recently collected samples. ANOVA was used to compare the mean estrogen and isoflavone concentrations in the urine samples (ANOVA: *P* > 0.05 for all analytes). The equality of variances for analytes in the archived samples and the recently collected samples was assessed by *F*-test (*P* > 0.05 for all analytes). Similarly, serum samples obtained within one week (n = 15) were simultaneously analyzed for E2. ANOVA and *F*-test showed no significant differences between recent and archived serum samples (*P* = 0.792 and 0.885, respectively). These results indicated that the analytes did not degrade in the archived samples.

Values under the detection limits were recorded as half of the detection limit. The urinary concentrations of the estrogens and isoflavones were normalized to urinary creatinine concentrations. Urinary creatinine concentration was assayed by using enzymatic method^34^. The individual differences were large for these biological concentrations, which ranged by several orders of magnitude. The concentrations were not normally distributed. To enable a statistical analysis comparison, the urinary and serum concentrations were transformed into common logarithmic values. The 4-OHE1/E1, 4-OHE2/E2, and E2/E1 ratios were also calculated to evaluate estrogen metabolism^35,36^. EQPs were defined according to the criteria proposed by Ideno et al. for postmenopausal and premenopausal women^37^, which are based on urinary daidzein and equol levels: log (equol/daidzein) ≥ − 1.42 indicates EQP status, and log (equol/daidzein) < − 1.42 indicates non-EQP status. Participants who had a progesterone concentration greater than 1 ng/mL were classified as the luteal-phase group.

### Statistical analysis

Differences in the means of potential outcome variables (log urinary and serum E2 levels) and proportions of other characteristics between the EQP and non-EQP groups were examined using Student’s *t*-test and Fisher’s exact test, respectively (two-tailed). The urinary equol excretion was evidently different between the EQP and non-EQP groups; thus, we could hypothesize that equol might affect E2 metabolism. The urinary equol concentrations were then used as the exposure variable (independent variable). To adjust the potential confounders, we conducted multivariable regression analyses. The analyses include a categorical variable (three smoking statuses and four menstrual statuses), and we employed analyses of covariance. Analyses of covariance were used to examine the correlation between urinary estrogen and equol levels, adjusted for age and smoking status and other variables. In a previous report, estrogen was associated with age and isoflavones, while smoking was also associated with estrogen and isoflavones^38^. Although an association between EQP statuses and overweight was controversial^39^, a report has shown a moderate correlation between BMI and urinary estrogens in postmenopausal Japanese women^40^. For these reasons, we chose them as covariates. Stepwise selection of variables was not employed to avoid overfitting^41^. We conducted stratified analyses for several variables (menopausal status, disease history, and menstrual cycle). This was because the potential effects of those stratified variables were not clear and modeling in the analysis of covariance was difficult, such as parameterization of variables, interaction terms thereof, and so on. Records with missing values were excluded from the analyses. JMP Pro (ver. 14; SAS Institute, Cary, NC) was used to perform these calculations. The alpha level for all tests was 0.05. P values were expressed with three significant figures unless they were less than 0.0001. We calculated the statistical power of the analysis of covariance using pwr.f2.test included in the R pwr package. When the effect size (Cohen’s f^2^) was 0.025 in the three-parameter model with 80% power (alpha = 0.05, two-tailed), the required sample size was 436. We analyzed a slightly larger number of samples to ensure statistical power.

## Results

### Characteristics and variables

Table 1 shows the characteristics of the study subjects and their urinary estrogen and isoflavone levels. The average age of the participants was 49.2 years (range: 23–74 years). Approximately half of the participants reported having regular menstrual cycles, and 11.6% were smokers. Some of the participants had current disease and/or a history of disease: 2 (present history: 1) reported endometriosis, 10 (2) myoma of the uterus, 1 (1) diabetes mellitus, 25 (11) thyroid disease, 5 (5) hypertension, 5 (2) cancer, and 76 (20) other diseases. In addition, 22 of the participants had multiple diseases: 18 had two diseases, and 4 had three diseases. Based on a cut-off level of log (equol/daidzein) ≥ − 1.42, 254 of the 520 participants (48.85%) were classified as EQPs. The participants were divided into EQP and non-EQP groups to evaluate possible differences in characteristics. Fisher’s exact test was used to examine differences in smoking, menstrual cycle, and disease history between the EQP and non-EQP groups. Smoking was significantly negatively associated with EQP status (*P* = 0.00152), as reported earlier for the same population^17^.

**Table 1:** Characteristics of the study subjects and their estrogen and isoflavone concentrations.

Urine samples	N^a^	Total (N = 520)	EQP (N = 254)	Non-EQP (N = 266)	*P* value
Age (yr)	520	49. 2 ± 10.1	49.5 ± 9.5	48.9 ± 10.7	0.532
Equol (μg/gCr)	520	77.6 (6.46)	269 (6.61)	24.5 (2.09)	** < 0.0001**^b^
Daidzein (μg/gCr)	520	1620 (4.57)	780 (5.25)	3240 (2.63)	** < 0.0001**^b^
Equol/Daidzein	520	0.0490 (9.33)	0.339 (3.89)	0.00759 (2.51)	** < 0.0001**^b^
Urinary E1 (μg/gCr)	376	2.95 (3.39)	3.09 (3.47)	2.88 (3.31)	0.538^b^
Urinary E2 (μg/gCr)	520	3.02 (3.63)	6.31 (3.89)	1.55 (2.09)	** < 0.0001**^b^
Urinary E3 (μg/gCr)	376	2.95 (3.80)	3.09 (3.89)	2.88 (3.72)	0.622^b^
Urinary 4-OHE1 (μg/gCr)	68	2.00 (3.98)	1.91 (3.98)	2.14 (3.98)	0.737^b^
Urinary 4-OHE2 (μg/gCr)	68	0.182 (3.47)	0.282 (4.57)	0.110 (1.62)	**0.00112**^b^
4-OHE1/E1	68	2.50 ± 5.78	2.60 ± 6.72	2.39 ± 4.62	0.883^b^
4-OHE2/E2	68	0.0721 ± 0.0433	0.0619 ± 0.0508	0.0836 ± 0.0299	**0.0385**^b^
E2/E1	376	4.08 ± 13.3	7.37 ± 18.5	0.932 ± 1.05	** < 0.0001**^b^
Urinary creatinine (g/L)	520	0.923 (1.80)	0.901 (1.90)	0.944 (1.71)	0.368
Menstrual cycle	507				0.182^c^
Regular cycles		240 (47.3%)	109 (43.8%)	131 (50.7%)	
Irregular cycles		43 (8.48%)	26 (10.4%)	17 (6.59%)	
Menopause		187 (36.9%)	98 (39.4%)	89 (34.5%)	
Experienced gynecological surgery		37 (7.30%)	16 (6.43%)	21 (8.14%)	
Smoking habit	501				**0.00152**^c^
Non-smoker		428 (85.5%)	221 (89.8%)	207 (81.2%)	
Current smoker		58 (11.6%)	16 (6.50%)	42 (16.5%)	
Ex-smoker		15 (2.99%)	9 (3.66%)	6 (2.35%)	
Disease history	520				1.00^c^
None		377(72.5%)	186(73.2%)	191(71.8%)	^c^
Current/past histories		143(27.5%)	68(26.8%)	75(28.2%)	^c^

Serum samples	N	Total (N = 100)	EQP (N = 57)	Non-EQP (N = 43)	*P* value
Age (yr)	100	49.1 ± 7.28	49.7 ± 6.94	48.3 ± 7.73	0.362^b^
Equol (μg/gCr)	100	30.2 (12.0)	102 (12.0)	6.03 (3.80)	** < 0.0001**^b^
Daidzein (μg/gCr)	100	524 (8.71)	186 (8.91)	2040 (3.24)	** < 0.0001**^b^
Serum E2 (pg/mL)	100	112 (1.35)	105 (1.04)	123 (1.05)	0.00517
Serum progesterone (ng/mL)	100	0.776 (2.34)	0.985 (1.12)	0.673 (1.14)	0.148^b^
Menstrual cycle	100				0.514^c^
Regular cycles		44 (44.0%)	25(43.9%)	19(44.2%)	
Irregular cycles		10 (10.0%)	4(7.02%)	6 (14.0%)	
Menopause		46(46.0%)	28 (49.1%)	18(41.9%)	
Smoking habit	100				0.833^c^
Non-smoker		84(84.0%)	49 (86.0%)	35 (81.4%)	
Current smoker		12(12.0%)	6 (10.5%)	6 (14.0%)	
Ex-smoker		4(4.0%)	2 (3.51%)	2 (4.65%)	

Values of urinary equol, daidzein, E1, E2, E3, 4-OHE1, 4-OHE2, creatinine, serum E2, and progesterone were geometric means and geometric standard deviations. Log: common logarithm; gCr: grams creatinine.

^a^The N for each item varies owing to missing values or selection for additional chemical analyses.

^b^*P* values indicates the results from two-tailed Student’s *t*-test between EQP and non-EQP groups. Log-transformed concentrations of urinary equol, daidzein, E1, E2, E3, 4-OHE1, 4-OHE2, creatinine, serum E2, and progesterone were used for this test.

^c^*P* values indicates the results from two-tailed Fisher’s exact test between EQP and non-EQP groups.

The urinary estrogen concentration is expressed as μg/gCr, as described previously^42^, and was further common log-transformed to normalize the distributions. The urinary concentration of the estrogen metabolite 4-OHE1 was typically higher than that of 4-OHE2 within total samples. Student’s *t*-test was used to examine differences in estrogen levels and isoflavone levels between the EQP and non-EQP groups. Regarding the isoflavone concentrations, the log equol (μg/gCr) and log equol/daidzein values were significantly higher in the EQP group than in the non-EQP group (*P* < 0.0001). Daidzein levels were lower in the EQP group (*P* < 0.0001), likely owing to the increased metabolism of daidzein to equol by the intestinal microflora.

Next, Student’s *t*-test was used to examine the urinary estrogen levels. Log urinary E2 (μg/gCr) was significantly elevated in the EQP group (*P* < 0.0001) compared with the non-EQP group, while the log urinary E1 and log urinary E3 levels did not differ between the groups. As a result, the E2/E1 ratio was also increased in the EQP group (*P* < 0.0001). Regarding estrogen metabolism, the log urinary 4-OHE2 value (μg/gCr) was significantly higher in the EQP group than in the non-EQP group (*P* = 0.00112), while the 4-OHE2/E2 ratio was lower in the EQP group (*P* = 0.0385). Log serum E2 (pg/mL) was significantly higher in the non-EQP group than in the EQP group (*P* = 0.00517).

Estrogen and isoflavone levels are also shown in each subgroup (Table S1). Age, menstrual cycle and smoking habits showed significant differences in urinary equol between subgroups (*P* = 0.0128, 0.0225 and 0.00577 by ANOVA, respectively). Urinary E2 levels were different among subgroups of smoking habits (*P* = 0.00179 by ANOVA). Hence, the effects of these variables were adjusted in following analyses.

### Analysis of the association between urinary estrogen and equol levels by analysis of covariance

The difference in urinary equol excretion between the EQP and non-EQP groups prompted us to hypothesize that equol could affect E2 metabolism. The urinary equol concentration was considered to be a more reliable indicator than EQP status. Smoking is significantly associated with log equol (μg/gCr), and age affects estrogen levels. Hence, analyses of covariance were employed to examine the correlation between urinary E2 and equol, including the effects of age and smoking (Table 2). Current smokers had lower urinary E2 than non-smokers (β = − 0.0908, *P* = 0.00985), and ex-smokers had higher E2 (β = 0.144, *P* = 0.00434). As in the bivariate analysis, the log equol value was significantly positively associated with the log urinary E2 (μg/gCr) value (β = 0.597, *P* < 0.0001), with an R^2^ value of 0.751. The parameter estimate corresponds to 5.74-fold change in urinary E2 by IQR increase in equol concentration (17.6 μg/gCr to 329 μg/gCr).

**Table 2:** Analysis of covariance of the association between urinary estradiol and equol levels.

Dependent variable: Log urinary E2 (μg/gCr)			
Independent variables	β	95% CI	*P*
Age (yr)	− 0.00154	− 0.00404–0.000965	0.222
Log Equol (μg/gCr)	0.597	0.566–0.628	** < 0.0001**
Smoking habit (current smoker)	− 0.0908	− 0.160 to − 0.0219	0.00985
(ex-smoker)	0.144	0.0452–0.242	0.00434

CI: confidence interval. Analysis of covariance was conducted for log urinary E2 as dependent variable with independent variables (log equol, age and smoking). Tobacco use was coded as 1 = non-smoker, 2 = current smoker, and 3 = ex-smoker. Model fitness: R^2^ = 0.751. IQR increase in equol (17.6 μg/gCr to 329 μg/gCr; 18.9-fold) was associated with 5.74-fold higher urinary E2 level.

Regarding the other urinary estrogens, log equol (μg/gCr) was significantly positively associated with log urinary 4-OHE2 (μg/gCr) value (β = 0.441, *P* < 0.0001) (Supplementary Table S2). After adjustment for the covariables (age and smoking), the log urinary E1 and log urinary 4-OHE1 (μg/gCr) became significant (β = 0.0697, *P* = 0.0277 and β = 0.197, *P* = 0.0209, respectively), but their R^2^ values were low (Supplementary Table S2).

Additional analyses were conducted to examine the influence of substituting values that were less than the limit of quantification (LOQ). Instead of substituting with half the LOQ, substituting with zero or with the LOQ was employed in extreme cases. The results showed similar trends as observed in the original analysis (Supplementary Table S3). The number of samples varied owing to the substitution, particularly when zero was substituted (logarithms were not applicable), but the effect sizes (beta parameter of equol) were almost the same as in the original analyses.

The association between EQP status and estrogens was also investigated by analysis of covariance, including age and smoking (Table 3, Supplementary Table S4). EQP status also showed a significant positive association with log urinary E2 (μg/gCr) (β = 0.299, *P* < 0.0001, R^2^ = 0.304) and log urinary 4-OHE2 (μg/gCr) (β = 0.233, *P* = 0.000435, R^2^ = 0.197).

**Table 3:** Analysis of covariance of urinary estradiol and equol producing status.

Dependent variable: Log urinary E2 (μg/gCr)			
Independent variables	β	95% CI	*P*
Age (yr)	0.00174	− 0.00243–0.00592	0.412
EQP	0.299	0.257–0.341	** < 0.0001**
Smoking habit (current smoker)	− 0.119	− 0.235 to − 0.00391	0.0428
(ex-smoker)	0.145	− 0.0197–0.311	0.0842

CI: confidence interval. Analysis of covariance was conducted for log urinary E2 as dependent variable with independent variables (EQP, age and smoking). Age and smoking were included as covariables in addition to EQP in all analyses. EQP status was coded as 1 = EQP and 0 = non-EQP; tobacco use was coded as 1 = non-smoker, 2 = current smoker, and 3 = ex-smoker. Model fitness: R^2^ = 0.304.

### Association between urinary equol and serum E2

To further examine the effect of increased excretion of E2 on serum E2 level, we analyzed an additional 100 paired serum and urine samples (Table 1). In the analysis of covariance, the log equol concentration was significantly negatively associated with the serum E2 levels (Table 4). However, the association was moderate (β = − 0.0225, *P* = 0.0462) compared with the association observed for urinary E2 excretion. IQR increase in urinary equol (4.51 μg/gCr to 189 μg/gCr) was associated with 0.919-fold lower serum E2 level.

**Table 4:** Analysis of covariance of the association between serum estradiol and urinary equol levels.

Dependent variable: Log serum E2 (pg/mL)			
Independent variables	β	95% CI	*P*
Age (yr)	− 0.00748	− 0.0108 to − 0.00413	** < 0.0001**
Log Equol (μg/gCr)	− 0.0225	− 0.0446 to − 0.000383	0.0462
Smoking habit (current smoker)	− 0.0260	− 0.0860–0.0340	0.391
(ex-smoker)	0.0160	− 0.0663–0.0983	0.701

CI: confidence interval. Analysis of covariance was conducted for log serum E2 as dependent variable with independent variables (log equol, age and smoking). Tobacco use was coded as 1 = non-smoker, 2 = current smoker, and 3 = ex-smoker. Model fitness, R^2^ = 0.233.

IQR increase in equol (4.51 μg/gCr to 189 μg/gCr; 41.8-fold) was associated with 0.919-fold lower serum E2 level.

### Verification of potential confounding effects by daidzein, menstrual status, and disease history

Equol levels are dependent on soy product consumption and the presence of intestinal bacteria. Urinary equol might be related to other factors that influence estrogen levels. To adjust for potential confounders related to soy intake, urinary daidzein levels were incorporated into the analysis of covariance model (Supplementary Table S5). As Supplementary Table S5 shows, the log equol (μg/gCr) value remained significantly positively associated with log urinary E2 (β = 0.599, *P* < 0.0001), even after adjustment based on the log daidzein value. It was possible that some potential EQPs were not identified because of a low soybean intake at the time of sampling, resulting in their misclassification as non-EQPs. To eliminate this possibility, those who had substantial soybean intake (urinary daidzein > 1000 μg/gCr, N = 327) were extracted. Analysis of covariance in the subpopulation showed comparable positive association between log urinary E2 and log urinary equol (β = 0.613, *P* < 0.0001, Supplementary Table S6).

The effect of equol on urinary E2 excretion may depend on menstrual status. Therefore, we performed a stratified analysis of premenopausal women and postmenopausal women (Supplementary Table S7). As shown in Supplementary Table S7, there was a positive association between log urinary equol and log urinary E2 levels in both premenopausal and postmenopausal women (β = 0.550, *P* < 0.0001; and β = 0.651, *P* < 0.0001, respectively). This analysis was also conducted for urinary E1 and E3, but there were no significant effects of equol on those excretions irrespectively of menstrual statuses (Supplementary Table S7).

Premenopausal women who were available for serum progesterone (N = 115) were classified into luteal phase (N = 63) and follicular phase (N = 52) groups based on the progesterone levels (Supplementary Table S8). There was a significant positive association between log urinary E2 and log equol in both groups (*P* < 0.0001 for both groups, Supplementary Table S8).

BMI has been suggested for association with estrogen metabolism^39^. In addition, previously, residential area was moderately associated with EQP statues^17^, and it could affect the association between urinary equol and E2. Effect of BMI on the association between urinary equol and E2 was examined among participants in one study area (N = 109, Supplementary Table S9). BMI was not associated with urinary E2 while log urinary equol concentration remained significant for urinary E2 (β = 0.631, *P* < 0.0001).

To eliminate possible bias from disease status on the association between the log urinary equol and log urinary E2 levels, analysis of covariance was performed for the participants without a self-reported history of disease (N = 363) (Supplementary Table S10). There was a significant positive association between the log equol and log urinary E2 values (β = 0.588, *P* < 0.0001), as was observed in the full dataset.

Finally, we conducted analysis of covariance for urinary E2 including all the potential factors (disease history, menstrual statuses, daidzein, smoking habit and age) (Table 5). The association between urinary E2 and equol was still significant (β = 0.602, *P* < 0.0001).

**Table 5:** Analysis of covariance of the association between log urinary E2 and log equol, including log daidzein, age, smoking, menstrual status and disease histories (N = 491).

Dependent variable: Log urinary E2 (μg/gCr)			
Independent variables	β	95% CI	*P*
Age (yr)	0.00244	− 0.00223–0.00712	0.306
Log Daidzein (μg/gCr)	− 0.0103	− 0.0488–0.0282	0.600
Log Equol (μg/gCr)	0.602	0.571–0.634	** < 0.0001**
Smoking habit (current smoker)	− 0.0901	− 0.161 to − 0.0199	0.0120
(ex-smoker)	0.142	0.0424–0.241	0.00528
Menstrual status (irregular cycles)	− 0.0448	− 0.117–0.0278	0.226
(menopause)	− 0.0406	− 0.0979–0.0167	0.164
(experienced gynecological surgeries)	0.0151	− 0.0725–0.103	0.734
Disease histories (without disease histories)	− 0.000352	− 0.0321–0.0314	0.982

CI: confidence interval. Analysis of covariance was conducted for log urinary E2 as dependent variable with independent variables (log equol, log daidzein, age, smoking, menstrual status and disease histories). Tobacco use was coded as 1 = non-smoker, 2 = current smoker, and 3 = ex-smoker. Menstrual status was coded as 1 = regular cycles, 2 = irregular cycles, 3 = menopause, and 4 = experienced gynecological surgery. Disease history was coded as 1 = person with current or past disease histories, and 2 = person without disease histories. Category 1 was set as a referent. Model fitness: R^2^ = 0.755.

## Discussion

In this observational study, urinary E2 excretion was more strongly positively associated with equol levels in the urine than other estrogens. This association was consistent with EQP status. The blood E2 levels were analyzed in this study, and there was a moderate negative association between serum E2 and urinary equol excretion. Physiologically, increased E2 excretion could correlate with decreased E2 levels in the blood. The majority of the E2 in the blood is bound to sex hormone-binding globulin (SHBG) and albumin, while a small portion of E2 is unbound and bioavailable. It has been previously shown that blood E2 levels are negatively associated with urinary equol levels in postmenopausal women^25^. However, we found that the concentration of daidzein, the precursor of equol, did not correlate with urinary E2 levels. This suggests that equol may play an important physiological role, and that equol-producing microflora may affect the risk of developing an estrogen-related disease. In addition, 4-OHE2 excretion was increased in EQPs. 4-OHE2 forms reactive quinone species and induces DNA damage^35^. Enhanced elimination of 4-OHE2 by equol may play a protective role in hormone-related carcinogenesis. On the other hand, as mentioned before, lowered estradiol levels in premenopausal women may affect their reproduction ability^29^. To verify the mechanism between equol and estrogens, intervention study will be needed to examine the causal relationship.

Although the mechanism by which equol affects E2 excretion was not investigated in this study, our findings still provide valuable insights. Urinary equol levels were more positively associated with urinary E2 and 4-OHE2 than with other estrogens. EQPs exhibited higher levels of E2 and 4-OHE2 excretion and lower 4-OHE2/E2 ratios compared with non-EQPs. Hence, 4-hydroxylation of E2 was not a dominant factor, but promoted clearance of E2 could be. Our findings demonstrated possible associations of varying strength between equol and urinary E2, 4-OHE2, and E1. Similarly, blood estrogens, and E2 in particular, bind to SHBG with different affinities^43^. Equol also binds to SHBG and non-competitively inhibits E2 binding to SHBG^44^. This could increase the fraction of free E2 in the blood, thereby making it available for metabolism^45^. These hypotheses should be investigated in future studies to provide biological evidence for the effects of equol. Effects of equol on estrogen syntheses such as 17beta-hydroxysteroid dehydrogenases (17β-HSD) or CYP19 aromatase are another possibility. However, in vitro study showed no significant effect of equol on aromatase activity^46^, and there has been no report on an inhibitory effect of equol on 17β-HSD^47^.

This was an observational study, and potential biases could have affected some of the associations that we observed. Our dataset did not include details of lifestyles, dietary habits, and BMI (except for one study area). These unmeasured variables may confound with urinary equol, E2 excretions and serum E2. While several studies have explored the relationship between various lifestyle factors and equol levels, few of these factors have been shown to have a significant association with urinary equol excretion. One exception is smoking, which is positively associated with EQP status^17^ and urinary E2^38^. Approximately 10% of the women included in this study smoked, which is consistent with a previously published national survey^48^. Multivariable analysis including smoking status showed consistent association between urinary E2 and equol. Therefore, the smoking characteristics of our study sample were standard. Nagata et al. demonstrated that women who consumed more dairy products had lower equol excretion than women who did not^49^. In addition, one report showed familial segregation of EQP, suggesting shared genetic or environmental factors within families^50^. Furthermore, equol levels are dependent on the levels of precursor isoflavones, i.e. soy consumption. The dietary habits of the participants were not recorded for this study. In particular, dairy products contain estrogens and account for 60–80% of dietary estrogen intake^51,52^. While it is possible that the relationship between EQP status and dairy consumption affects estrogen levels in humans, our findings suggest that this is not likely, because, if EQPs consumed fewer dairy products, their dietary estrogen intake and urinary estrogen excretion would be lower than those of non-EQPs. The effects of soy consumption were also evaluated using an analysis of covariance model and stratified analysis (Table5, Supplementary Table S5 and S6), which showed that daidzein did not affect the association between equol and E2 excretion. In addition, after menopause, blood estrogen levels decrease significantly. Age does not influence EQP status, and in a subgroup analysis of postmenopausal women and premenopausal women, the association between E2 and equol was unchanged. In relation to menstrual status, in premenopausal women, regularity and length of menstrual cycle can vary among individuals. In this study, samples from premenopausal women were divided into luteal and follicular phase groups; the associations between urinary E2 and equol were similar in both groups. But in future study, equol effects on menstrual cycle via FSH and LH^53^ are worth investigation. Finally, disease status may modify estrogen levels because of the pathophysiology of the disease or treatment. However, analysis of samples from women with and without a history of disease showed similar associations (Supplementary Table S10). Indeed, while it is likely that multiple, as yet unidentified, factors determine EQP status, approximately 50% of the participants in this study were EQPs, which is consistent with previous reports^54^, suggesting that there was no selection bias in our study population. Furthermore, the observed association between urinary E2 and equol remained high even after adjustment for several variables, so any unidentified confounder, if it exists, would have to be strongly associated with urinary E2. Nonetheless, future study with comprehensive background information (BMI, food consumption, etc.) can help the confirmation of this finding.

In this study, urine samples were used to assess isoflavone levels. Equol excretion might be promoted by kidney conditions and affect E2 levels in urine. Generally, the level of chemicals in the urine varies owing to dilution, and normalizing to creatinine levels is not always an effective approach. Although consumption of soy products may differ from day to day, the biological half-life of equol is ca. 8 h, and equol concentrations in the urine are strongly correlated with blood equol concentrations^10^. Hence, urinary equol level is considered a valid biomarker to distinguish with non-EQP and EQP. On the other hand, biological half-life of daidzein is shorter than that of equol and urinary daidzein would partially reflect soy product consumption^13,55^. Nevertheless, presence of daidzein in urine indicated short-term exposure to soy products, and influence on direct interaction for equol-E2 association is less likely. However, detecting equol in the urine is difficult if people do not consume soybean products. Our full sample set analysis might underestimate the proportion of potential EQPs. The stratified analysis according to urinary daidzein concentration produced similar results as the total sample analysis (Supplementary Table S6). Therefore, the definition of EQP status based on urinary equol and daidzein concentrations is unlikely to affect the association between urinary E2 and equol.

The urinary E2 levels were analyzed in all participants, as the primary outcome in this study was urinary E2 excretion, but the levels of other estrogens were also analyzed in selected participants. Serum samples were analyzed in 100 participants. Thus, the statistical power differed among the target analytes, which may have affected the results. However, urinary E1, 4-OHE1, and E3 levels were very similar between EQPs and non-EQPs, and analyzing more samples would not have changed the results. EQPs were defined by the equol/daidzein ratio in the urine^34^, as equol concentration is affected by intake of its precursor, daidzein. Using a different definition of EQP may have resulted in different associations with estrogens. The analysis of covariance model showed that urinary E2 levels correlated with urinary equol levels in a dose-dependent manner. In addition, E1 and 4-OHE1 levels showed statistically significant associations with urinary equol levels, even in a limited number of samples (Supplementary Table S2). Their β parameters and model fitness values were lower than those of E2 or 4-OHE2, indicating that E2 is the primary target of the effects of equol. The further studies are needed to clear the causal effect of equol with internal E2 levels.

In conclusion, our findings suggest that equol may promote urinary E2 excretion and modify moderately blood E2 levels in women.

## Supplementary Information

Supplementary Information.

## Data Availability

All data generated or analyzed during this study are included in this published article.
