# Supplementary material for: Urinary equol levels are positively associated with urinary estradiol excretion in women
Source: Sci Rep. 2021 Sep 30;11:19532. doi: 10.1038/s41598-021-98872-2 (PMC8484452; doi:10.1038/s41598-021-98872-2)
Supplement: Supplementary file 1 — Supplementary Information. [file 41598_2021_98872_MOESM1_ESM.pdf]

Supplementary information

Urinary equol levels are positively associated with urinary estradiol excretion in women.

Tomoko Fujitani [1], Yukiko Fujii [2], Zhaoqing Lyu [1], Mariko Harada Sassa [1], Kouji H. Harada [1,*]

[1]Department of Health and Environmental Sciences, Kyoto University Graduate School of Medicine, Kyoto, 606-8501 Japan

[2]Department of Pharmaceutical Sciences, Daiichi University of Pharmacy, Fukuoka 815−8511, Japan

[*]Correspondence: kharada-hes@umin.ac.jp; Tel.: +81-75-753-4490 (K.H.H.)

This file includes:

Supplementary Table S1. Characteristics of the study subjects by subgroups and their urinary estrogen and isoflavone concentrations.
Supplementary Table S2. Analysis of covariance for the association between urinary estrogen and equol levels.
Supplementary Table S3. Analysis of covariance of association between urinary estradiol and urinary equol levels with different substitutions for values less than detection limits.
Supplementary Table S4. Analysis of covariance for association between equol producing status and urinary estrogens.
Supplementary Table S5. Analysis of covariance of the association between log urinary E2 and log equol, including log daidzein, age, and smoking (N = 501).
Supplementary TableS6. Analysis of covariance of the association between urinary equol and estradiol levels in women who had high-daidzein levels (>1000 μg/gCr, N=327).
Supplementary Table S7. Analysis of covariance of the association between urinary equol and estrogen levels in premenopausal and postmenopausal women.
Supplementary Table S8. Analysis of covariance of the association between urinary equol and estradiol levels stratified to luteal phase women and follicular phase women (N=115).
Supplementary Table S9. Analysis of covariance of the association between log urinary E2 and log equol, including log daidzein, age, smoking, BMI, and disease histories among

study subjects from Kyoto (N = 109).

Supplementary Table S10. Analysis of covariance of the association between urinary estradiol and equol levels in women without a history of disease (N=363).

Supplementary Fig. S1. Typical chromatogram of analysis of phytoestrogens and estrogens.

	Urinary E2 (μg/gCr)	Equol (μg/gCr)	Urinary creatinine (g/L)
Age			
20-39	2.48 (3.27)	52.5 (5.93)	1.14 (1.80)
40-59	3.40 (3.84)	94.1 (7.04)	0.915 (1.78)
60-	2.99 (3.54)	81.1 (5.46)	0.753 (1.73)
	P=0.0704	*P*=0.0128	*P*<0.0001
Menstrual cycle			
Regular cycles	2.85 (3.39)	60.6 (6.24)	1.07 (1.80)
Irregular cycles	3.40 (4.07)	117 (7.26)	0.906 (1.87)
Menopause	3.19 (3.84)	99.4(6.46)	0.793(1.70)
Experienced gynecological surgery	3.28 (3.95)	80.7 (6.12)	0.812 (1.95)
	P=0.717	*P*=0.0225	*P*<0.0001
Smoking habit			
Non-smoker	3.21 (3.59)	86.0 (6.44)	0.914 (1.77)
Current smoker	1.82 (3.13)	37.4 (5.19)	1.12 (1.79)
Ex-smoker	5.23 (5.47)	88.9 (9.15)	0.830 (2.42)
	P=0.00179	*P*=0.00577	*P*=0.0309
Disease history			
None	3.08 (3.63)	80.6 (6.61)	0.951 (1.79)
Current/past histories	2.95 (3.67)	73.0 (6.07)	0.855 (1.83)
	P= 0.725	*P*= 0.589	*P*= 0.0685

Supplementary Table S1. Geometric means of urinary estradiol, equol and creatinine concentrations of the study subjects by subgroups. Value of E2, Equol, and creatinine was geometric mean and geometric standard deviations. gCr: grams creatinine. † *P* values indicates the results from analysis of variance for log-transformed concentrations of urinary equol, E2, creatinine.

**Dependent variables**	**N**	**Log Equol (µg/gCr) as independent variable**			**Model fitness**
		β	95% CI	*P* value	R^2
Log urinary E1 (µg/gCr)	365	0.0697	0.0670–0.132	0.0277	0.185
Log urinary E3 (µg/gCr)	365	0.0590	−0.00489–0.123	0.0702	0.275
Log urinary 4-OHE1 (µg/gCr)	66	0.197	0.0309–0.363	0.0209	0.0972
Log urinary 4-OHE2 (µg/gCr)	66	0.441	0.332–0.551	<0.0001	0.523

Supplementary Table S2. Analysis of covariance for the association between urinary estrogen and equol levels. CI: confidence interval. Analysis of covariance was conducted for each log urinary estrogen as dependent variable with independent variables (log equol, age and smoking). Age and smoking were included as covariables. Tobacco use was coded as 1 = non-smoker, 2 = current smoker, and 3 = ex-smoker.

**Dependent variables**	**N**	**Independent variable: Log urinary Equol (μg/gCr)**			**Model fitness**
		β	95% CI	*P* value	R^2
BLOQ assumed as 0					
Log urinary E1 (μg/gCr)	105	−0.0397	−0.164–0.0849	0.529	0.00747
Log urinary E2 (μg/gCr)	177	0.575	0.509–0.641	<0.0001	0.645
Log urinary E3 (μg/gCr)	99	−0.0407	−0.172–0.0906	0.540	0.193
Log urinary 4OH-E1 (μg/gCr)	12	−0.165	−0.520–0.191	0.316	0.641
Log urinary 4OH-E2 (μg/gCr)	12	0.494	−0.250–1.24	0.160	0.552
BLOQ assumed as LOQ					
Log urinary E1 (μg/gCr)	365	0.0825	0.0208–0.144	0.00897	0.144
Log urinary E2 (μg/gCr)	501	0.583	0.555–0.611	<0.0001	0.781
Log urinary E3 (μg/gCr)	365	0.0662	0.00261–0.130	0.0414	0.237
Log urinary 4OH-E1 (μg/gCr)	66	0.207	0.0517–0.361	0.00977	0.123
Log urinary 4OH-E2 (μg/gCr)	66	0.438	0.335–0.540	<0.0001	0.555

Supplementary Table S3. Analysis of covariance for association between urinary estrogens and urinary equol levels with different substitutions for values less than detection limits. BLOQ: below limits of quantification. CI: confidence interval. Urinary equol levels less than LOQ were substituted to zero or LOQ values. Analysis of covariance was conducted for each log urinary estrogen as dependent variable with independent variables (log equol, age and smoking). Age and smoking were included as covariables in addition to EQP in all analyses. EQP status was coded as 1 = EQP and 0 = non-EQP; tobacco use was coded as 1 = non-smoker, 2 = current smoker, and 3 = ex-smoker.

Dependent variables	N	Independent variable: EQP			Model fitness
		β	95% CI	*P* value	R^2
Log urinary E1 (μg/gCr)	365	0.00798	−0.0428–0.0587	0.757	0.174
Log urinary E3 (μg/gCr)	365	−0.000170	−0.0524–0.0520	0.995	0.268
Log urinary 4-OHE1 (μg/gCr)	66	−0.0203	−0.173–0.133	0.791	0.0151
Log urinary 4-OHE2 (μg/gCr)	66	0.233	0.108–0.358	0.000435	0.197

Supplementary Table S4. Analysis of covariance for association between equol producing status and urinary estrogens. CI: confidence interval. Analysis of covariance was conducted for each log urinary estrogen as dependent variable with independent variables (log equol, age and smoking). Age and smoking were included as covariables in addition to EQP in all analyses. EQP status was coded as 1 = EQP and 0 = non-EQP; tobacco use was coded as 1 = non-smoker, 2 = current smoker, and 3 = ex-smoker.

Dependent variable: Log urinary E2 (μg/gCr)			
**Independent variables**	β	95% CI	*P* value
Age (yr)	−0.00145	−0.00397–0.00107	0.258
Log Daidzein (μg/gCr)	−0.0121	−0.0501–0.0260	0.533
Log Equol (μg/gCr)	0.599	0.567–0.630	<0.0001
Smoking habit			
(current smoker)	−0.0896	−0.159 to −0.0206	0.0110
(ex-smoker)	0.142	0.0435–0.241	0.0048

Supplementary Table S5. Analysis of covariance of the association between log urinary E2 and log equol, including log daidzein, age, and smoking (N = 501). CI: confidence interval. Analysis of covariance was conducted for log urinary E2 as dependent variable with independent variables (log equol, log daidzein, age and smoking). Log daidzein, age, and smoking were included as covariables in addition to log equol in this analysis. Tobacco use was coded as 1 = non-smoker, 2 = current smoker, and 3 = ex-smoker. Model fitness: $R^2 = 0.752$.

Dependent variable: Log Urinary E2 (µg/gCr)			
**Independent Variables**	β	95% CI	*P* value
Age	−0.00159	−0.00451–0.00133	0.285
Log Equol (µg/gCr)	0.613	0.578–0.648	<0.0001
Smoking habit			
(current smoker)	−0.0798	−0.165–0.00575	0.0674
(ex-smoker)	0.150	0.0214–0.278	0.0223

Supplementary Table S6. Analysis of covariance of the association between urinary equol and estradiol levels in women who had high-daidzein levels (>1000 µg/gCr, N=327). CI: confidence interval. Analysis of covariance was conducted for log urinary E2 as dependent variable with independent variables (log equol, age and smoking). Age and smoking were included as covariables in addition to log equol. Tobacco use was coded as 1 = non-smoker, 2 = current smoker, and 3 = ex-smoker. Model fitness: R^2 =0.797.

**Dependent variable: Log urinary E2 (μg/gCr)**			
**Premenopausal (N=234, average age=40.42 ± 5.81, median age=39)**			
**Independent variables**	β	95% CI	*P* value
Age (yr)	0.00364	−0.00304–0.0103	0.284
Log Equol (μg/gCr)	0.550	0.501–0.598	<0.0001
Smoking habit			
(current smoker)	−0.0633	−0.155–0.0284	0.175
(ex-smoker)	0.0651	−0.0689–0.199	0.340
**Postmenopausal (N =181, average age =57.91 ± 4.66, median age=59)**			
**Independent variables**	β	95% CI	*P* value
Age (yr)	0.00338	−0.00487–0.0116	0.420
Log Equol (μg/gCr)	0.651	0.603–0.698	<0.0001
Smoking habit			
(current smoker)	−0.046	−0.204–0.112	0.566
(ex-smoker)	0.0802	−0.168–0.328	0.524

**Dependent variable: Log urinary E1 (μg/gCr)**			
Premenopausal (N = 172, average age = 40.41 ± 5.27, median age = 39)			
Independent variables	β	95% CI	*P* value
Age	−0.00994	−0.0208–0.000872	0.0713
Log Equol (μg/gCr)	0.0361	−0.0335–0.106	0.307
Smoking habit			
(ex-smoker)	0.112	−0.0807–0.306	0.252
(current smoker)	−0.0644	−0.196–0.0675	0.336
Postmenopausal (N =133, average age =58.09 ± 4.9, median age = 59)			
Independent variables	β	95% CI	*P* value
Age	0.00602	−0.0124–0.0244	0.519
Log Equol (μg/gCr)	0.0914	−0.0267–0.210	0.128
Smoking habit			
(ex-smoker)	−0.171	−0.868–0.527	0.629
(current smoker)	0.153	−0.256–0.562	0.460
**Dependent variable: Log urinary E3 (μg/gCr)**			
Premenopausal (N = 172, average age = 40.41 ± 5.27, median age = 39)			

Independent variables	β	95% CI	*P* value
Age	−0.00544	−0.0189–0.00799	0.425
Log Equol (μg/gCr)	0.0445	−0.0420–0.131	0.311
Smoking habit			
(ex-smoker)	0.142	−0.0978–0.382	0.244
(current smoker)	−0.0678	−0.232–0.0960	0.415
Postmenopausal (N =133, average age =58.09 ± 4.90, median age = 59)			
Independent variables	β	95% CI	*P* value
Age	−0.0135	−0.0291–0.00212	0.0896
Log Equol (μg/gCr)	0.0842	−0.0159–0.184	0.0986
Smoking habit			
(ex-smoker)	0.688	0.096–1.279	0.023
(current smoker)	−0.184	−0.531–0.162	0.294

Supplementary Table S7. Analysis of covariance of the association between urinary equol and estrogen levels in premenopausal and postmenopausal women. CI: confidence interval. Participants with irregular menstrual cycles or who had experienced gynecological surgery were excluded. Analysis of covariance was conducted for log urinary E2, E1 and E3 as dependent variables with independent variables (log equol, age and smoking). Tobacco use was coded as 1 = non-smoker, 2 = current smoker, and 3 = ex-smoker. In estradiol analysis, model fitness was R^2 = 0.704 for premenopausal women; R^2 = 0.810 for postmenopausal women. In estrone analysis, R^2 =0.0337 for premenopausal women; R^2 = 0.0242 for postmenopausal women. In estriol analysis, R^2 = 0.0185 for premenopausal women; R^2 =0.119 for postmenopausal women.

Dependent variable: Log urinary E2 (μg/gCr)			
**Luteal phase (N=63, average age=41.90 ± 5.58, median age=42)**			
**Independent variables**	β	95% CI	*P* value
Age (yr)	−0.0128	−0.0328–0.00716	0.204
Log Equol (μg/gCr)	0.271	0.145–0.397	<0.0001
Smoking habit			
(current smoker)	−0.00233	−0.302–0.298	0.988
(ex-smoker)	0.0783	−0.355–0.512	0.719
**Follicular phase (N =52, average age =43.81 ± 5.68, median age=43)**			
**Independent variables**	β	95% CI	*P* value
Age (yr)	0.000174	−0.0201–0.0205	0.986
Log Equol (μg/gCr)	0.246	0.144–0.347	<0.0001
Smoking habit			
(current smoker)	0.0498	−0.219–0.319	0.712
(ex-smoker)	−0.117	−0.434–0.201	0.463

Supplementary Table S8. Analysis of covariance of the association between urinary equol and estradiol levels stratified to luteal phase women and follicular phase women (N=115). Premenopausal participants who had progesterone concentration over 1 ng/mL were classified to luteal phase group. CI: confidence interval. Participants with irregular menstrual cycles or who had experienced gynecological surgery were excluded. Analysis of covariance was conducted for log urinary E2 as dependent variable with independent variables (log equol, age and smoking). Age and smoking were included as covariables in addition to log equol. Tobacco use was coded as 1 = non-smoker, 2 = current smoker, and 3 = ex-smoker. Model fitness: R^2 = 0.261 for luteal phase women; R^2 = 0.365 for follicular phase women.

Dependent variable: Log urinary E2 (μg/gCr)			
**Independent variables**	β	95% CI	*P* value
BMI	−0.00116	−0.0164–0.0140	0.880
Age (yr)	−0.00819	−0.0216–0.00522	0.228
Log Daidzein (μg/gCr)	0.00669	−0.0752–0.0886	0.872
Log Equol (μg/gCr)	0.631	0.567–0.695	< 0.0001
Smoking habit			
(current smoker)	−0.131	−0.348–0.0864	0.235
(ex-smoker)	0.294	−0.0726–0.661	0.115
Menstrual status			
(irregular cycles)	−0.0510	−0.237–0.135	0.588
(menopause)	0.0308	−0.125–0.186	0.695
(experienced gynecological surgeries)	0.0210	−0.293–0.336	0.895
Disease histories			
(without disease histories)	−0.00820	−0.0705–0.0541	0.794

Supplementary Table S9. Analysis of covariance of the association between log urinary E2 and log equol, including log daidzein, age, smoking, BMI and disease histories among study subjects from Kyoto (N = 109). CI: confidence interval. Analysis of covariance was conducted for log urinary E2 as dependent variable with independent variables (log equol, log daidzein, age and smoking). Log daidzein, age, and smoking were included as covariables in addition to log equol in this analysis. Tobacco use was coded as 1 = non-smoker, 2 = current smoker, and 3 = ex-smoker. Menstrual status was coded as 1= regular cycles, 2 = irregular cycles, 3 = menopause, and 4 = experienced gynecological surgery. Disease history was coded as 1 = person with current or past disease histories, and 2 = person without disease histories. Category 1 was set as a referent. Model fitness: R^2 = 0.806.

Dependent variable: Log urinary E2 (μg/gCr)			
**Independent variables**	β	95% CI	*P* value
Age (yr)	−0.00237	−0.00560–0.000867	0.1510
Log Equol (μg/gCr)	0.588	0.551–0.625	<0.0001
Smoking habit			
(current smoker)	−0.0562	−0.139–0.0262	0.181
(ex-smoker)	0.0831	−0.0378–0.204	0.177

Supplementary Table S10. Analysis of covariance of the association between urinary estradiol and equol levels in women without a history of disease (N=363). CI: confidence interval. Analysis of covariance was conducted for log urinary E2 as dependent variable with independent variables (log equol, age and smoking). Tobacco use was coded as 1 = non-smoker, 2 = current smoker, and 3 = ex-smoker. R^2 = 0.734.

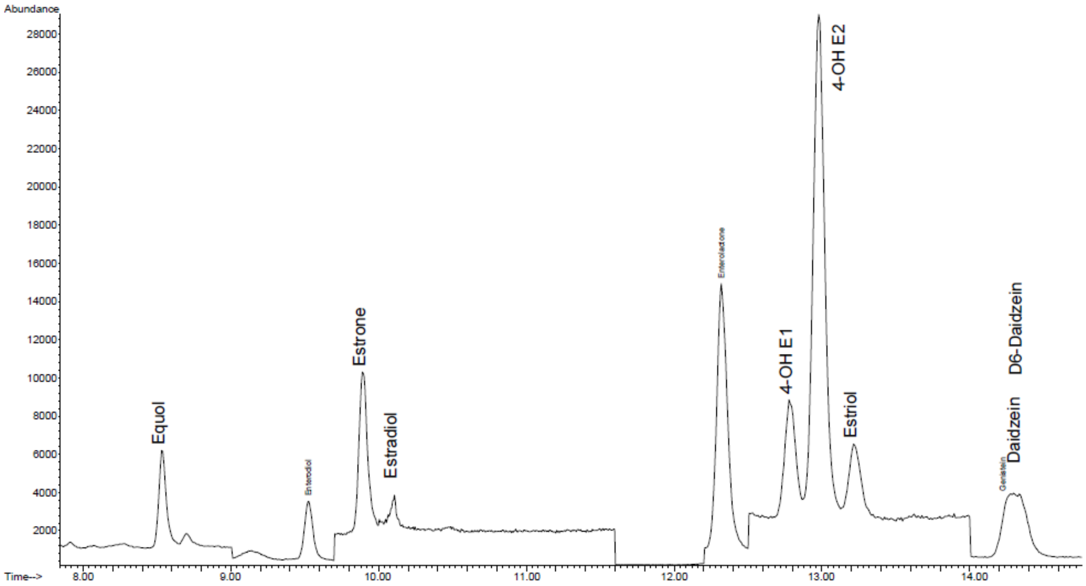

Fig. S1. Typical chromatogram of analysis of phytoestrogens and estrogens.
